# Deployment, suicide, and overdose among comorbidity phenotypes following mild traumatic brain injury: A retrospective cohort study from the Chronic Effects of Neurotrauma Consortium

**DOI:** 10.1371/journal.pone.0222674

**Published:** 2019-09-20

**Authors:** Mary Jo Pugh, Alicia A. Swan, Megan E. Amuan, Blessen C. Eapen, Carlos A. Jaramillo, Roxana Delgado, David F. Tate, Kristine Yaffe, Chen-Pin Wang

**Affiliations:** 1 VA Salt Lake City Health Care System, Informatics, Decision-Enhancement and Analytic Sciences Center, Salt Lake City, Utah, United States of America; 2 Department of Internal Medicine, University of Utah, School of Medicine, Salt Lake City, Utah, United States of America; 3 Department of Psychology, University of Texas at San Antonio, San Antonio, Texas, United States of America; 4 Department of Physical Medicine and Rehabilitation, VA Greater Los Angeles Health Care System, Los Angeles, California, United States of America; 5 Department of Medicine, David Geffen School of Medicine at UCLA, Los Angeles, California, United States of America; 6 Polytrauma Rehabilitation Center, South Texas Veterans Health Care System, San Antonio, Texas, United States of America; 7 Missouri Institute of Mental Health, University of Missouri-St. Louis, St. Louis, Missouri, United States of America; 8 Department of Psychiatry, Neurology & Epidemiology, University of California, San Francisco, San Francisco, California, United States of America; 9 Department of Epidemiology and Biostatistics, University of Texas Health Science Center at San Antonio, San Antonio, Texas, United States of America; Duke University School of Medicine, UNITED STATES

## Abstract

Mild traumatic brain injury in the Veteran population is frequently comorbid with pain, post-traumatic stress disorder, and/or depression. However, not everyone exposed to mild traumatic brain injury experiences these comorbidities and it is unclear what factors contribute to this variability. The objective of this study was to identify comorbidity phenotypes among Post-9/11 deployed Veterans with no or mild traumatic brain injury and examine the association of comorbidity phenotypes with adverse outcomes. We found that Veterans with mild traumatic brain injury (n = 93,003) and no brain injury (n = 434,378) were mean age of 32.0 (SD 9.21) on entering Department of Veterans Health Administration care, were predominantly Caucasian non-Hispanic (64.69%), and served in the Army (61.31%). Latent class analysis revealed five phenotypes in each subcohort; Moderately Healthy and Mental Health phenotypes were common to both. The Healthy phenotype was found only in no brain injury. Unique phenotypes in mild traumatic brain injury included Moderately Healthy+Decline, Polytrauma, and Polytrauma+Improvement. There was substantial variation in adverse outcomes. The Polytrauma+Improvement phenotype had the lowest likelihood of adverse outcomes. There were no differences between Moderately Healthy+Decline and Polytrauma phenotypes. Phenotypes of comorbidity vary significantly by traumatic brain injury status including divergence in phenotypes (and outcomes) over time in the mild traumatic brain injury subcohort. Understanding risk factors for the divergence between Polytrauma vs. Polytrauma+Improvement and Moderately Healthy vs. Moderately Healthy+Decline, will improve our ability to proactively mitigate risk, better understand the early patterns of comorbidity that are associated with neurodegenerative sequelae following mild traumatic brain injury, and plan more patient-centered care.

## Introduction

Caring for Post-9/11 Veterans with mild traumatic brain injury (mTBI) has vexed clinicians, because these patients’ complex patterns of comorbidity complicate diagnosis and treatment. Although most individuals with mTBI experience a complete resolution of symptoms within three months of injury, an estimated 15% report persistent post-concussive symptoms long after exposure [1,2]. This group, often called the ‘miserable minority’ [3], can present a year or more after mTBI exposure with continued complex symptomology. Their comorbidities can include post-traumatic stress disorder (PTSD), chronic pain, and depression. Different combinations of these conditions have been referred to as the Polytrauma Clinical Triad (TBI, post-traumatic stress disorder and pain [4–6]) or the Deployment Trauma Phenotype (PTSD, depression and mTBI) [7,8]. Identifying and characterizing comorbidity phenotypes among Post-9/11 Veterans with mTBI is essential for directing clinical care to prevent adverse outcomes. However, cross-sectional approaches are limited in their ability to reveal the emergence of comorbidity phenotypes and their evolution over time.

In a heterogeneous longitudinal cohort of Post-9/11 Veterans, latent class analysis (LCA) identified the accumulation of chronic disease, TBI, and post-concussive conditions as five comorbidity phenotypes that were consistent across men and women: Healthy, Chronic Disease, Mental Health, Pain, and Polytrauma. Two additional phenotypes were found in men:emerging chronic disease and Polytrauma+Chronic Disease [5]. Veterans with diagnoses of TBI were found among several of the groups, but were heavily clustered in the Polytrauma comorbidity phenotype. The Polytrauma phenotype was associated with high levels of self-reported depression, PTSD, and pain symptoms; high rates of unemployment and somatization, and low evaluations of self-rated health, family function, social support and community reintegration [9, 10]. However, Veterans diagnosed with TBI were also scattered among other phenotypes with more positive outcomes [5]. This finding suggests that a variety of comorbidity phenotypes exist among Veterans with TBI. However, these initial investigations used a heterogeneous Post-9/11 Veteran sample, and included all TBI severities, which may have obscured the characterization of comorbidity phenotypes among Veterans with TBI.

Because the majority of TBIs are mild [11], we sought to understand comorbidity phenotypes in TBI with a focus on mTBI. The purpose of this project was to describe longitudinal comorbidity phenotypes among Post-9/11 Veterans with mTBI in contrast to those with no evidence of TBI. We hypothesized that specific comorbidity phenotypes would be revealed among those with mTBI that were distinct from those among the Veterans with no diagnosis of TBI. Moreover, we hypothesized that among those with mTBI, comorbidity phenotypes characterized by complex comorbidity that include mental health, substance use disorder, and pain would be associated with higher risk for suicide-related behavior (SRB; suicidal ideation/attempt), overdose, homelessness, and mortality.

## Materials and methods

### Study cohort

As part of the Chronic Effects of Neurotrauma Consortium (CENC) epidemiology study, we identified electronic health record data in the Department of Veteran’s Affairs Veterans Health Administration (VHA) databases for Veterans deployed in support of Post-9/11 contingencies including Operations Enduring Freedom, Iraqi Freedom, and New Dawn (OEF/OIF/OND). To examine long-term patterns of mTBI-related comorbidities, we included individuals who a) entered VHA care between October 1, 2001 and September 30, 2011, b) received five or more years of VHA care (inpatient, outpatient, or pharmacy) before September 30, 2014, and c) received at least one year of care after 2007, when the VHA implemented mandatory TBI screening. We further restricted this analysis to individuals who met criteria for mTBI or had no indication of TBI as described below. This study received institutional review board approval from the University of Texas Health Science Center at San Antonio, the University of Utah, the Bedford VHA Hospital, and the Department of Defense (DoD) Human Research Protection Office, with a waiver of informed consent.

### Data sources

We acquired administrative data from both the VHA and DoD. VHA data sources included inpatient and outpatient data from the national repository in Austin, TX, the VHA pharmacy benefits management outpatient dataset, data from the Corporate Data Warehouse, the TBI screening and comprehensive TBI evaluation (CTBIE) datasets from the Office of Patient Care Services, and mortality data from the VHA vital status files. DoD data included the DoD Trauma Registry (DoDTR). We merged these datasets using an encrypted identifier to create a longitudinal record for each Veteran.

### Measures

#### TBI severity

**Fig 1:**
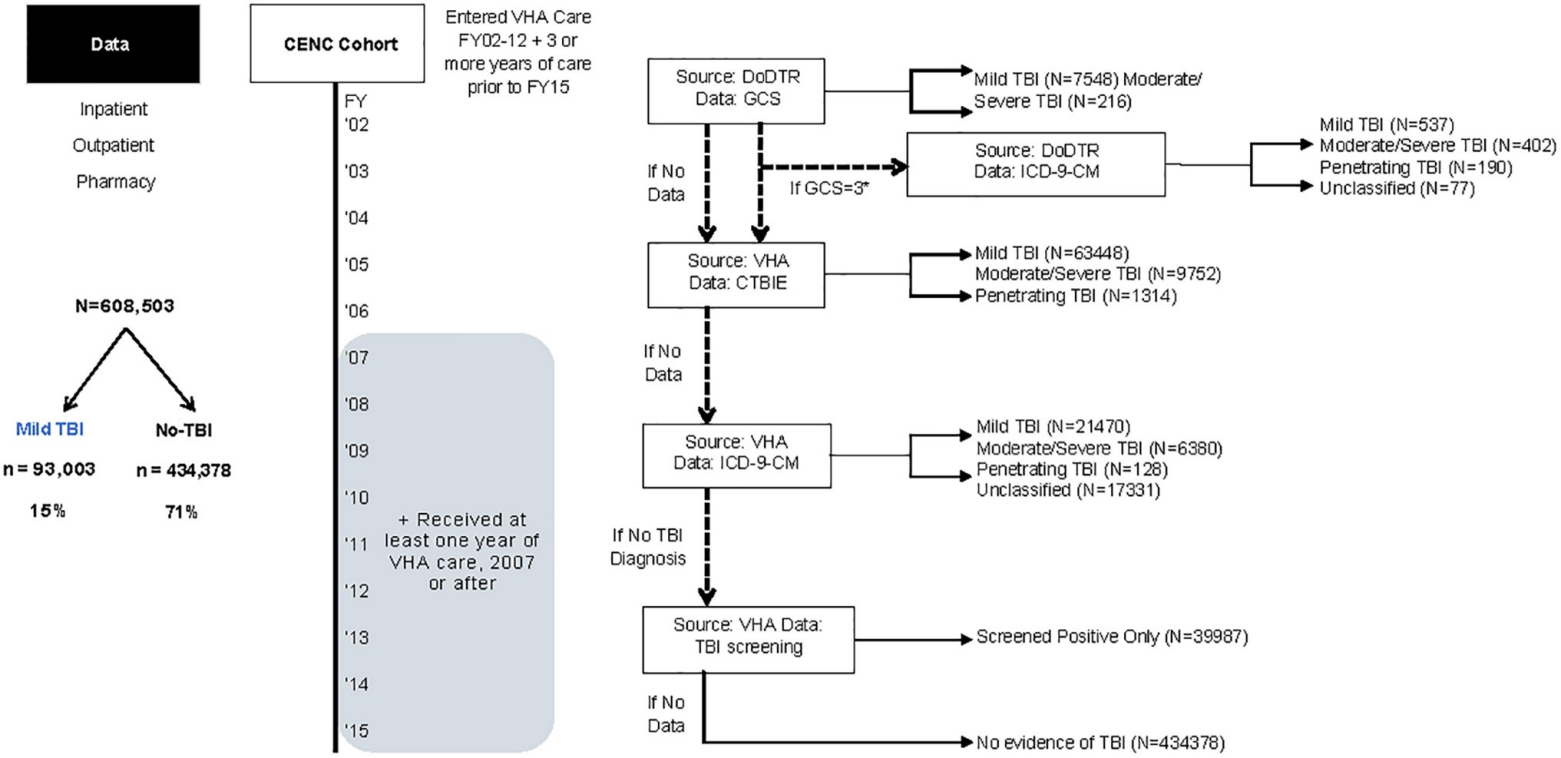
Cohort of Post-9/11 Veterans in VHA care and TBI severity algorithm. Graphic representation of the selection of the Post-9/11 cohort and the algorithm used to categorize traumatic brain injury (TBI) severity using a hierarchical selection from among the data sources. In this algorithm, data from the Department of Defense trauma registry (DoDTR) was first considered. The DoDTR includes Glasgow Coma Scale scores, which range between 3 and 15. An individual with a score of 12 or less would be considered to have a Moderate/Severe TBI, whereas an individual with a score of 13 or more would be considered to have mTBI. Then, if DoDTR data was not available, self-reported duration of loss of consciousness, alteration of consciousness, and post-traumatic amnesia from the comprehensive TBI evaluation (CTBIE) was used to classify TBI severity based on Department of Defense criteria [12]. Then, if that data were also not available, International Classification of Diseases, Ninth Revision, Clinical Modification (ICD-9-CM) diagnosis codes from VHA care were used to classify TBI severity based on coding guidance from the Department of Defense [13]. If a Veteran did not have evidence among any of these data sources to determine exposure to TBI, they were categorized as No-TBI.

We identified TBI severity using an algorithm that incorporated the DoDTR and VHA administrative data sources (Fig 1). Individuals who met criteria for mTBI and those who had no indication of TBI exposure (hereafter, No-TBI) were included in this analysis as a comparator to those with mTBI.

#### Diagnosed health conditions

We used International Classification of Diseases, 9th revision, Clinical Modification (ICD-9-CM) diagnosis codes from inpatient and outpatient data (excluding ancillary and telephone care) to identify comorbidities associated with mental health, post-concussive symptoms, pain, and other conditions associated with sleep and obesity (see supplemental table “S1 Table”). We created dichotomous variables (0 = no diagnosis; 1 = one or more diagnoses) for each condition during each year of the study period.

In addition to conditions used to identify comorbidity phenotypes, we also identified adverse events during the period of comorbidity trajectory development. Suicide related behavior (SRB) and overdose were defined using ICD-9-CM diagnosis codes and homelessness using ICD-9-CM diagnostic codes and clinic codes (“S1 Table”) [14,15]. Each condition is represented as a dichotomous variable (0 = not present; 1 = present) for use as a covariate in outcomes analyses.

#### Demographic characteristics

We obtained military and socio-demographic characteristics from VHA administrative data. Socio-demographic characteristics included age at entry to VHA care, sex, race/ethnicity, marital status, and percent service-connected disability (see Table 1). Military characteristics included branch of service, rank, military component, and multiple deployments. Individuals for whom the date of the last deployment was later than the date of first deployment were identified as having multiple deployments.

**Table 1:** Baseline characteristics of the Post-9/11 Veteran cohort stratified by TBI status during Year 1 of VHA care.

	Mild TBI N (%)	No-TBI N (%)	Chi Square P-value
**SOCIO-DEMOGRAPHICS**	n = 93,003	n = 434,378	<.001
**Age** (Mean [SD])	29.79 [7.8]	32.53 [9.4]	
**Sex:** Female	5608 (6.03)	67,425 (15.52)	
**Race/Ethnicity**			
Caucasian Non-Hispanic	64,724 (69.59)	276,429 (63.64)	<.001
African American	12,334 (13.26)	82,788 (19.06)	
Hispanic	11,671 (12.55)	50,312 (11.58)	
Asian	1980 (2.13)	12,220 (2.81)	
Native American/Pacific Islander	1591 (1.71)	5693 (1.31)	
Unknown	703 (0.76)	6936 (1.60)	
**Marital Status:** Married	38,406 (41.30)	198,535 (45.71)	
**Service Connected Disability** (Median)	80	60	
**First year of VHA**^a^ **Care** (Median)	2008	2008	
**MILITARY CHARACTERISTICS**			
**Rank:** Enlisted	89.784 (96.54)	397,813 (91.58)	<.001
**Branch**			
Army	67,109 (72.16)	256,239 (58.99)	
Marine Corps	16,945 (18.22)	52,313 (12.04)	
Navy/Coast Guard	5504 (5.92)	66,678 (15.35)	
Air Force	3445 (3.70)	59,148 (13.62)	
**Component:** Active Duty	61,012 (65.60)	249,408 (57.42)	
**Multiple Deployments**	51,025 (54.86)	226,354 (52.11)	
**COMORBID CONDITIONS**			
Depression	20,547 (22.09)	48,608 (11.19)	<.001
Substance Use Disorder	11,168 (12.01)	23,899 (5.50)	
Posttraumatic Stress Disorder	34,979 (37.61)	48,608 (11.19)	
Anxiety	12,922 (13.89)	31,416 (7.23)	<.001 <.001
Tinnitus	14,140 (15.20)	31,703 (7.30)	
Hearing Loss	14,197 (15.27)	36,873 (8.49)	
Vestibular Dysfunction	2509 (2.70)	2796 (0.64)	
Blurred vision	2561 (2.75)	2645 (0.61)	
Blindness	1053 (1.13)	2022 (0.47)	
Seizure	716 (0.77)	1040 (0.24)	
Cognitive Dysfunction	7225 (7.77)	1607 (0.37)	
Stroke/ Transient Ischemic Attack	345 (0.37)	540 (0.12)	
Headache	18,929 (20.35)	23,922 (5.51)	
Neck Pain	5916 (6.36)	13,660 (3.14)	
Back Pain	25,255 (27.16)	76,515 (17.61)	
Other Pain (arthritis/musculoskeletal)	19,631 (21.11)	66,457 (15.30)	
Obesity	5854 (6.29)	29,342 (6.75)	
Obstructive Sleep Apnea	1038 (1.12)	3767 (0.87)	
Insomnia	9267 (9.96)	15,894 (3.66)	
Hypersomnia	498 (0.54)	1594 (0.37)	
Pituitary conditions	53 (0.06)	202 (0.05)	0.19
**ADVERSE EVENTS (Trajectory Development Period)**			
Suicide Related Behavior	6179 (6.64)	10,272 (2.36)	<.001
Overdose	4485 (4.82)	8360 (1.92)	
Homelessness	9770 (10.51)	23,132 (5.33)	
**ADVERSE OUTCOMES: (VHA Care Year 6 +)**			
Suicide Related Behavior	3803 (4.09)	5903 (1.36)	<.001
Overdose	2773 (2.98)	4958 (1.14)	
Homelessness	6782 (7.29)	16,121 (3.71)	
Mortality	1305 (1.40)	4241 (0.98)	

^a^Abbreviations: VHA, Veterans Health Administration

#### Adverse outcomes

We used the same ICD-9-CM codes described above to identified adverse outcomes of SRB, overdose, and homelessness in year six and beyond. SRB, overdose and homelessness were evaluated through September 30, 2015 to ensure consistency in identification due to implementation of ICD-10; mortality was evaluated through September 30, 2017.

### Analysis

We first examined frequencies of each diagnosed condition in the study cohort during the first five years of VHA care and conducted latent class analysis (LCA) to identify distinct comorbidity trajectory classes stratified by mTBI and No-TBI [16,17]. LCA is a statistical modeling technique used to identify distinct unobserved subgroups within a population. In-depth information about the modeling technique is provided elsewhere [5].

We evaluated LCA models that included 3–8 latent classes and selected the final model based on goodness of fit (Bayesian information criteria and Akaike information criteria). We then labeled each latent class (hereafter comorbidity phenotype) based on the clinical pattern of comorbidities with the highest probabilities at baseline and at year 5. Individuals were assigned a comorbidity phenotype using the pseudoclass technique [18]. Chi-square tests were used to compare patient characteristics between comorbidity classes for mTBI and No-TBI groups. A priori levels of significance were p-value less than 0.01. Hypothesis tests were two-sided. We performed the analyses using SAS 9.3 and Mplus 7.4.

Finally, we used logistic regression models to examine the extent to which comorbidity phenotypes predicted adverse outcomes (SRB, overdose, homelessness, and mortality). To determine the extent to which comorbidity phenotypes enhance the prediction of these outcomes, we conducted nested logistic regression analyses for each outcome measure using two models. The first was a reduced model that included only socio-demographic and military characteristics and covariates (prior SRB, overdose, homelessness) as predictors. The second model added phenotypes to the reduced model. We then compared model fit based on area under the receiver operating curve (ROC) for the two logistic regression models to determine if inclusion of comorbidity phenotypes improved the model fit and prediction of adverse outcomes [19].

## Results and discussion

Of the 608,516 individuals who met the overall cohort inclusion criteria, 434,378 (71%) had No-TBI and 93,003 (15%) met criteria for mTBI. Table 1 shows socio-demographics, military characteristics, comorbid conditions, and rates of adverse outcomes for those with mTBI and No-TBI. Briefly, individuals with mTBI were significantly more likely to be younger, male, Caucasian non-Hispanic, and have a higher percentage of service-connected disability. They were also significantly less likely to have most recently served in the National Guard, Reserve or Army. They were also significantly less likely to have been an Officer or Warrant Officer. Finally, Veterans with mTBI were also significantly more likely than those with No-TBI to have diagnoses of mental health disorders, post-concussive symptoms, pain, and sleep problems (Table 1).

### Comorbidity phenotypes identified in this Post-9/11 Veteran cohort

**Fig 2:**
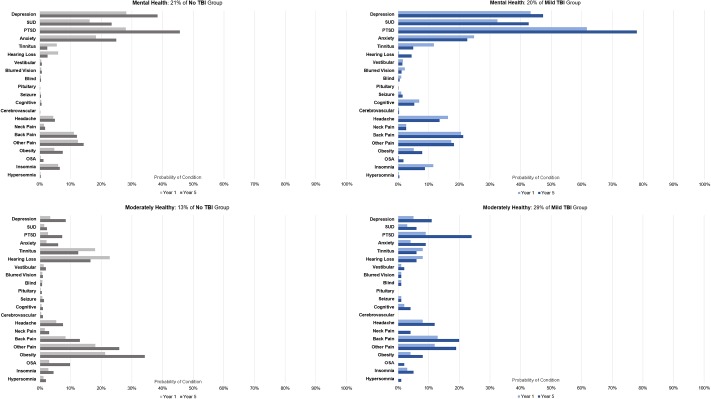
Comorbidity phenotypes common to No-TBI and mTBI cohorts. Figure shows the probability that an individual within a phenotype was diagnosed with each condition in year 1 and year 5 of VHA care.

The stratified LCA revealed five comorbidity phenotypes in both the No-TBI and mTBI subcohorts. Both subcohorts included a phenotype characterized by having relatively low probabilities of back pain, other pain, mental health and sensory conditions compared to the base-rate in the population (Moderately Healthy: MoH), and another characterized by increasing probabilities of mental health conditions including PTSD, depression and substance use disorder by year five (Mental Health) (Fig 2). Similar patterns of diagnosis probabilities were exhibited by these two phenotypes among each of the mTBI and No-TBI subcohorts, however, the MoH phenotype was far more common in the mTBI subcohort (29%) compared to No-TBI (13%). Notably, in the mTBI subcohort, the Mental Health phenotype had significantly higher probabilities for depression, substance use disorder, PTSD and anxiety (p<.001) than the No-TBI subcohort (all p<.01).

**Fig 3:**
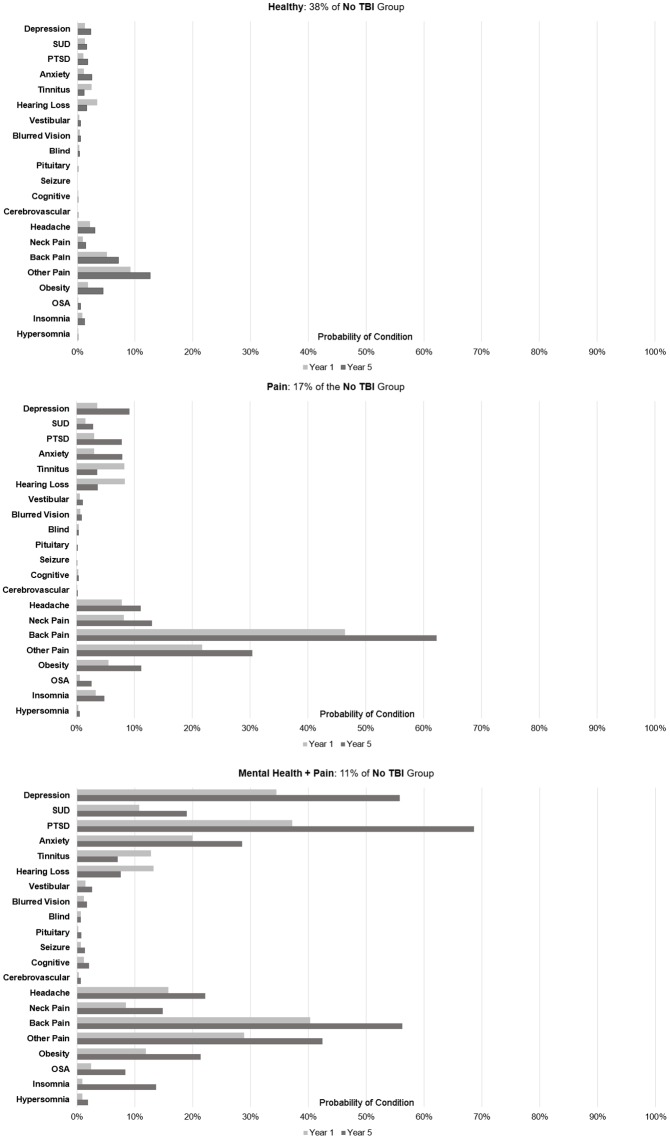
Distinct comorbidity phenotypes in No-TBI cohorts. Figure shows the probability that an individual within a phenotype was diagnosed with each condition in year 1 and year 5 of VHA care.

The No-TBI subcohort also included comorbidity phenotypes with a) a very low probability of any diagnoses throughout the trajectory development period (Healthy), b) a high probability of back pain and other [musculoskeletal] pain (Pain), and c) a high probability of both mental health and pain-related conditions (Mental Health+Pain) (Fig 3).

**Fig 4:**
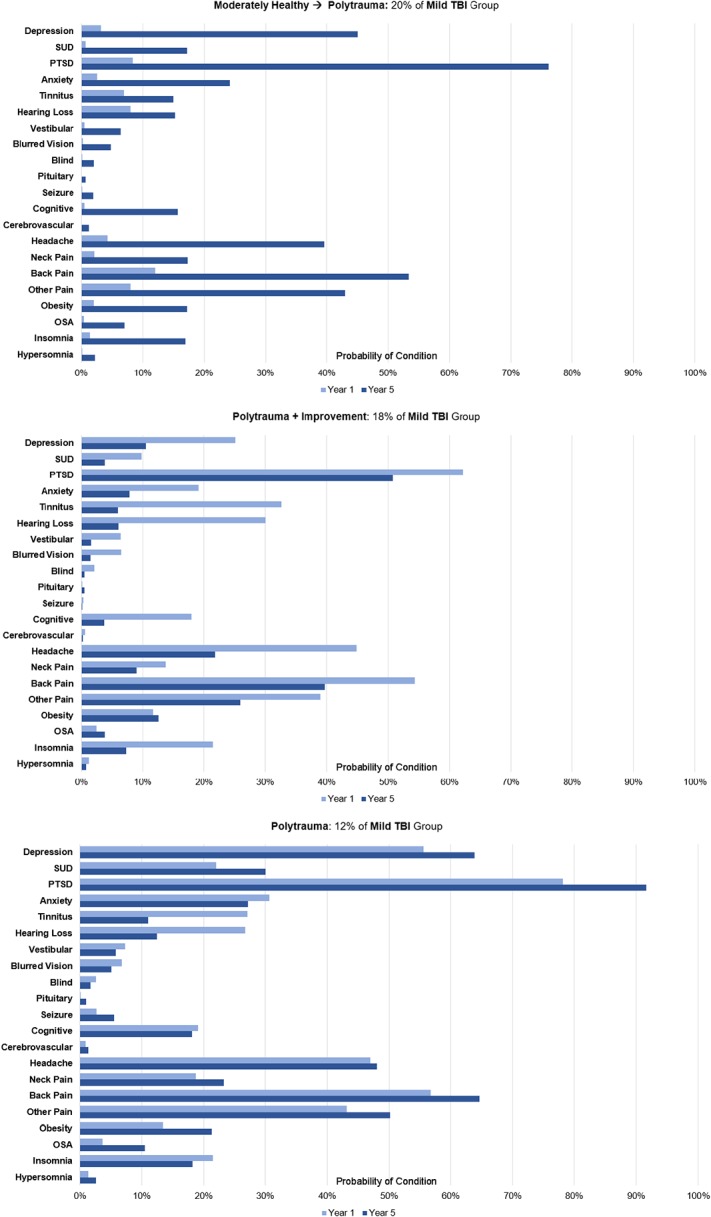
Distinct comorbidity phenotypes in mTBI cohorts. Figure shows the probability that an individual within a phenotype was diagnosed with each condition in year 1 and year 5 of VHA care.

The three additional phenotypes for those with mTBI included MoH and Polytrauma phenotypes, some of which transitioned to decline or improvement. MoH+Decline included individuals who began much like the aforementioned MoH but subsequently demonstrated significant decline (higher probabilities of mental health diagnoses, post-concussion symptoms, or pain) by year five. The Polytrauma phenotype had high probabilities of mental health disorders, post-concussion symptoms and pain consistently throughout the trajectory development period. The Polytrauma+Improvement phenotype started with characteristics similar to the Polytrauma phenotype but had substantially reduced probabilities of pain, post-concussive symptoms, and mental health conditions by year five (Fig 4). The most notable differences among comorbidity phenotypes in the mTBI subcohort were that relative to the MoH phenotype, MoH+Decline were less likely to be in the National Guard/Reserve (34% vs. 43%, p<.01). Further, individuals in the Polytrauma+Improvement phenotype were more likely to have multiple deployments compared to the Polytrauma phenotype (56% vs. 50%, p<.01).

### Adverse outcomes

**Fig 5:**
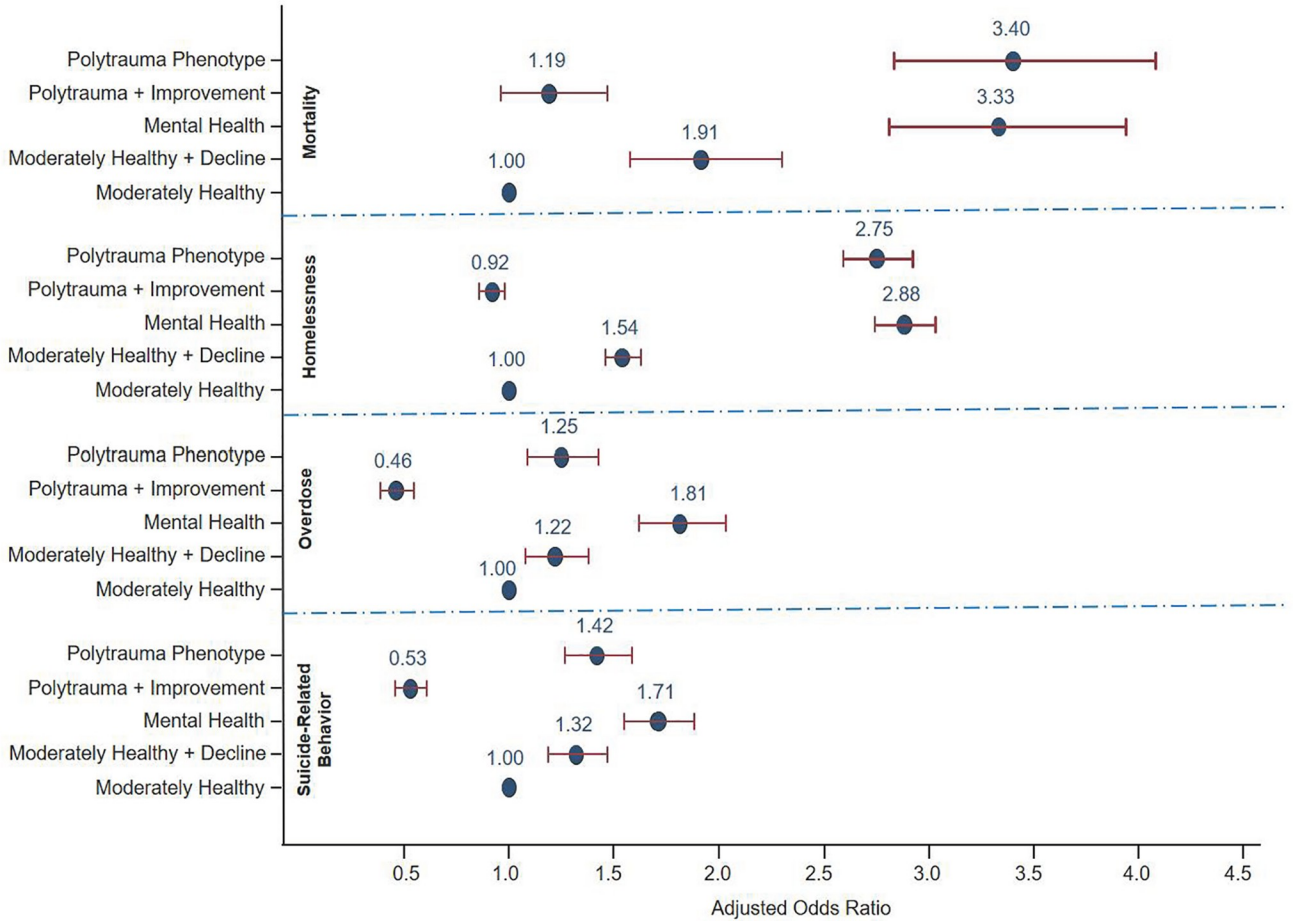
Logistic regression analyses predicting adverse outcomes by comorbidity phenotype. Odds ratios adjusted for socio-demographic/ military characteristics and corresponding adverse event described in Table 1.

“S2 Table” shows the prevalence of SRB, overdose, homelessness and mortality by comorbidity phenotype. For SRB, overdose and homelessness the highest and lowest prevalence consistently occurred in the Mental Health and Polytrauma+Improvement phenotypes respectively. Fig 5 shows the adjusted odds ratios associated with the comorbidity phenotype from each of the logistic regression analyses predicting SRB, overdose, homelessness, and mortality. Because MoH is the “healthiest” phenotype, it was used as the reference group. These odds ratios were adjusted for demographic characteristics, and diagnosis of the adverse event of interest during the trajectory development period.

The Polytrauma+Improvement phenotype was significantly less likely to have SRB, overdose and homelessness compared with the MoH group. Both the MoH+Decline and Polytrauma phenotypes had significantly higher odds than the MoH phenotype for SRB, overdose and mortality. Finally, the Mental Health phenotype had significantly higher odds of all adverse outcomes relative to the MoH phenotype. Comparison of the fully adjusted and reduced models for each outcome revealed that the addition of comorbidity phenotype significantly enhanced the model prediction.

## Discussion

This study is the first to compare the accumulation of comorbidity among Veterans with mTBI relative to Veterans with no diagnosis of TBI, and adds to growing knowledge about the unique combination of post-deployment conditions experienced by Post-9/11 Veterans. Our TBI-stratified analyses revealed five comorbidity phenotypes among each of the mTBI and No-TBI subcohorts. Of those five phenotypes, two were common to each of these subcohorts: 1) moderately healthy (probabilities of the assessed comorbidities significantly lower than the base-rate for the population), and 2) mental health comorbidity (high probabilities for mental health conditions). Moreover, specific comorbidity phenotypes were significantly associated with adverse outcomes after the trajectory development period, even after controlling for diagnoses those adverse outcomes during the trajectory development period. The addition of comorbidity phenotypes to the regression analyses significantly enhanced the prediction of these adverse events, suggesting that the identification of comorbidity phenotypes may be a useful marker in identifying individuals at risk for suicide, overdose, homelessness and mortality in the course of clinical care and may provide an important opportunity to prevent these potentially catastrophic outcomes.

The comorbidity phenotypes that emerged among the No-TBI cohort were similar to those in a prior cross-sectional analysis of the broader Post-9/11 Veteran population in VHA care, except that there was no Polytrauma phenotype [5]. This finding validates this approach, as one would not expect a phenotype that emphasizes post-concussive symptoms or TBI-related comorbidity in a cohort of Veterans without evidence of TBI.

Unlike our previous analyses of comorbidity phenotypes in a Post-9/11 Veteran cohort, this analysis among those with mTBI identified phenotypes with both significant decline (MoH+Decline) and significant improvement (Polytrauma+Improvement) over the course of the trajectory development period. This suggests that the derivation of comorbidity phenotypes among more homogeneous subgroups, such as those associated with mTBI, may provide a more comprehensive understanding of changes in health status over time in subpopulations. Further evaluation of comorbidity phenotypes that begin as characteristically similar but diverge by the end of the trajectory development period (i.e., MoH vs. MoH+Decline and Polytrauma vs. Polytrauma+Improvement) may be critical to understanding the care needed by this cohort of Veterans. Early indicators that predict adverse outcomes are needed, and identifying them would help mobilize appropriate clinical services and support networks for survivors of TBI and their support network. The distinct comorbidity phenotypes identified in this study may be unique to Post-9/11 Veterans, which would likely impact policy regarding informal caregiving that is available to Post-9/11 Veterans with mental health conditions and mTBI [20].

Differences among these phenotypes may be related to resilience or other premorbid protective psychological characteristics, an idea enforced by the finding that the Polytrauma+Improvement phenotype was more likely to have multiple deployments [21]. Alternatively, emergence of different phenotypes over time may be related to treatment approaches. Though the identification of treatment(s) that may have led to positive changes in health status is beyond the scope of the current paper, it is possible that successful pain treatments may have reduced overall mental health symptomology and improved sleep function among those in the Polytrauma+Improvement phenotype, which could promote optimal cognitive function. However, differences may also be attributed to variability in exposures experienced while in military service, genetics, and/or biomarkers that are not currently available.

An additional finding of interest was that individuals in the Mental Health phenotype were more likely than those in the Polytrauma phenotype to experience subsequent overdose and homelessness. In contrast, one might expect these outcomes to be equally or more common in the more medically complex Polytrauma phenotype where pain is also more prevalent [22, 23]. Examination of the probabilities of conditions between the two phenotypes, however, revealed significantly higher probabilities of SUD in both years one (37% vs. 19%) and five (41% vs. 22%) of the trajectory development period for Mental Health vs. Polytrauma phenotypes. This higher probability of comorbid SUD and mental health diagnoses (i.e., dual diagnosis) is likely a substantial factor in differential outcomes, particularly overdose and homelessness, which are commonly linked to SUD and dual diagnosis [23–25]. Systematic screening for and treatment of SUD among individuals with mood disorders and PTSD may be one way to mitigate risks of overdose and homelessness in this population. It is also possible that mental health diagnoses take clinical priority during the early years of care and that pain conditions existed, but were of a lesser severity or were being clinically addressed outside the VHA system or through self-care or self-medication [26].

Despite being a comprehensive evaluation of the longitudinal ascertainment of comorbidity phenotypes, this work has limitations inherent in the use of administrative data. First, while some errors in diagnostic coding may occur, it is unlikely that coding errors would be more likely in the mTBI cohort. In fact, the integration of TBI-relevant data from the DoDTR, CTBIE and ICD-9-CM diagnostic codes from VHA care likely improves the accuracy of identifying mTBI. Next, information on comorbidity is based on health care Veterans seek within the VHA system and does not take into account care received from other health systems. Because all deployed Post-9/11 Veterans receive five years of free care after discharge or demobilization, access to care is equal for individuals regardless of TBI status, which limits bias among those included in these cohorts. Finally, our analyses of comorbidity phenotypes were based on VHA health system data after service and did not include comorbidity identified during military service. Finally, our findings may not generalize to Post-9/11 Veterans who do not seek VA care.

Our findings of comorbidity phenotypes among Post-9/11 Veterans with mTBI, and the ability of these phenotypes to predict adverse events above and beyond traditional covariates, suggests that additional work is important to delineate phenotype emergence in Veterans who enter VHA care. Future work will benefit from examining additional expanded DoD and VHA longitudinal health system data and other data types including bio specimens and diagnostic imaging. The incorporation of this data in deep learning models may enhance precision medicine by providing clinicians with information on the optimal treatment for a specific patient’s phenotype.

## Supporting information

S1 Table Diagnostic code definitions for the development of comorbidity phenotypes and adverse events among Post-9/11 Veterans. The ICD-9-CM codes for each condition used in the latent class model are provided here.
(DOCX)

S2 Table Prevalence of adverse outcomes by comorbidity phenotypes: Mild traumatic brain injury. This table provides the number (and percentage) of individuals in each comorbidity phenotype who were subsequently diagnosed with suicide related behavior, overdose, or homelessness or who died after the phenotype development period.
(DOCX)
