# Supplementary material for: Deployment, suicide, and overdose among comorbidity phenotypes following mild traumatic brain injury: A retrospective cohort study from the Chronic Effects of Neurotrauma Consortium
Source: PLoS One. 2019 Sep 20;14(9):e0222674. doi: 10.1371/journal.pone.0222674 (PMC6754132; doi:10.1371/journal.pone.0222674)
Supplement: S2 Table — This table provides the number (and percentage) of individuals in each comorbidity phenotype who were subsequently diagnosed with suicide related behavior, overdose, or homelessness or who died after the phenotype development period. (DOCX) [file pone.0222674.s002.docx]

**S2 Table: Prevalence of Adverse Outcomes by Comorbidity Phenotypes: Mild Traumatic Brain Injury**

|  | **Moderately Healthy**  **N (%)**  29,168  (31.35%) | **Moderately Healthy + Decline**  **N (%)**  17,706 (19.04%) | **Mental Health**  **N (%)**  28,268 (19.64%) | **Polytrauma + Improvement**  **N (%)**  16,538  (17.78%) | **Polytrauma**  **N (%)**  11,333  (12.19%) |
| --- | --- | --- | --- | --- | --- |
| **Suicide Related Behavior N (%)** | 833 (2.86) | 769 (4.34) | 1259 (6.89) | 255 (1.54) | 687 (6.06) |
| **Overdose N (%)** | 598 (2.05) | 494 (2.79) | 1082 (5.92) | 162 (0.98) | 437 (3.86) |
| **Homelessness N (%)** | 3082 (10.57) | 2702 (15.26) | 4788 (26.21) | 1584 (9.58) | 2694 (23.77) |
| **Mortality N (%)** | 207 (0.71) | 242 (1.37) | 434 (2.38) | 143 (0.86) | 279 (2.46) |
